# Case of Cancer of the Stomach with Tubercular Peritonitis and Dropsy of the Peritoneum, Necroscopy, and Remarks

**Published:** 1848-12

**Authors:** Lawrence Turnbull

**Affiliations:** Philadelphia


*Case of Cancer of the Stomach with Tubercular Peritonitis and Dropsy of the Peritoneum, Necroscopy, and Remarks.* By LAWRENCE TURNBULL, M. D., Philadelphia.

Charles Howison, an artist, thirty-nine years of age, of ordinary height, very thin, intelligent, with intemperate habits.

His diet has been sufficiently good; diathesis strumous; several members of his family having died of phthisis pulmonalis.

He has suffered from pain in the abdomen and weakness of the back for four years; with constant constipation, and the generation of large quantities of gaseous matter in stomach and intestines.

In the month of July, 1846, he immigrated to this country from Edinburgh, (Scotland,) and settled in Brooklyn, New York, and was somewhat benefited by the sea voyage. But a short time after his arrival the pain returned, and also the constipation, for which, considering it to be of a bilious nature, he used powerful purgative medicines. This constant purgation produced hemorrhoids, which gave him great uneasiness.

Several months after his arrival in this city, his disease had very much increased, from his sedentary habits and constant effort to support himself and a sister, who depend upon him.

His bowels were often confined for weeks, and his stomach rejected all kinds of nourishment. He had constant vomiting for three months; at first once a day, but afterwards more frequently, and his emaciation had become considerable, although he was never remarkable for "embonpoint." He now applied for medical advice, as an out-door patient of the Philadelphia Dispensary, and continued to walk thither for some time. The physician in attendance treated him for dyspepsia, and, so difficult was it to overcome the constipation, that he took at one dose six compound cathartic pills, and one ounce of castor oil; and this even failed to excite the peristaltic action of the bowels.

He now became so weak as to be unable to walk as far as the Dispensary; his friends therefore called in a physician of his neighbourhood, who prescribed a bran diet and small doses of P. Carb. Lig., but these he was unable to retain. Upon making a more careful examination, he found a dropsical effusion. He then prescribed a mixture of ol. terebinth. and oleum ricini, equal parts; but he could not take the nauseous dose, and the pain produced by it being so intense as to make him cry out with agony. The physician finding he had refused his prescriptions, discontinued his attendance. Prof. J. K. Mitchell, (as one of the physicians of St. Andrew Society,) now visited him, and found him suffering from retention of urine, but being prevented from repeating his visit on account of indisposition, he requested me to see him.

Actual state on my visiting him, Sunday, October 24th, 1847. The patient appeared lively, and altogether rational; sense of sight almost lost; face of a peculiar straw coloured tint; tongue moist and of a bright red colour, with dark brown patches posteriorly; a great desire for cooling drinks, with constant vomiting of dark grumous looking matter.

The abdomen very flat, slightly enlarged over the pubic region, with cicatrices from blisters over the epigastric region. Percussion and auscultation over the heart and lungs reveal the normal physical signs. Skin dry and soft, pulse one hundred and four, with pain over the liver upon pressure. Has passed his urine by drops for twenty-four hours, and suffers much pain from distension of the bladder.

Wishing to give him as little pain as possible, I allowed him to inhale the vapour of sulphuric ether from a sponge for a few seconds, not to narcotize him, but to allay spasm. It had the happiest effect; he did not suffer the slightest pain from the introduction of a silver catheter, and the removal of forty-eight ounces of urine of a dark and sanguineous appearance. After allowing it to stand for twenty-four hours, it deposited no sediment; and neither heat nor nitric acid coagulated it, showing the absence of albumen.

It effervesced with the acid, showing an alkaline nature. He could not lie in bed from the impediment to the action of the diaphragm, and therefore sat up in a chair, night and day. The lower extremities were discoloured and swollen.

Monday, the 25th Oct.—Skin pale and moist; did not sleep well, and suffered with hiccough, which was relieved by the use of a mixture of equal parts of brandy and spt. ether sulph. c. Pulse one hundred, weak and thread-like. Introduced the catheter, and removed eighteen ounces of urine of the same colour as before. Eyes much congested, and bowels not free. Prescribed essence of beef and iced brandy, with enemata.

Evening of Monday.—Hectic fever: pulse over one hundred, with much pain in the eyes, burning in the throat, and heat in stomach; discontinued the essence of beef. Solut. morph. sulph. ℥js. A teaspoonful every hour until sleep is induced, with small pieces of ice to allay thirst.

Tuesday, Oct. 26.—Slept part of the night, under the influence of the morphia; has had involuntary discharge of fæces. Pulse reduced to eighty; teeth and gums covered with sordes; suffers from constant vomiting of particles of dark looking matter like disorganised mucous membrane. Removed a few ounces of urine of a lighter colour. Brandy and water and ice, and continue the morphia at night.

Wednesday, Oct. 27.—Low muttering delirium: not able to distinguish his friends; pulse seventy-four; fluid increasing in the cavity; more difficulty in introducing the catheter, having to place him in a much more recumbent position; removed about twenty ounces of urine, of a still lighter colour, with strong ammoniacal odour. Continued the treatment.

Thursday, Oct. 28, 11 o'clock, A. M. Pulse sixty-five; delirium still continues, with subsultus; almost moribund; removed twenty-four ounces of urine. He continued in this state until half past six, P. M., when he died.

*Necroscopy.*—Twenty-four hours' after death; atmosphere dry; walls of abdomen near the umbilicus of a livid hue; brain not examined.

Chest: the heart and pericardium perfectly natural, with the usual quantity of fluid The aorta filled with a yellowish white mass of coagula. There was no adhesion of the lungs to the pleura, but they were covered all over with spots of a dark colour, of different sizes, and which cut with difficulty. The bronchial tubes were apparently healthy.

Abdomen: stomach bound down by bands of lymph, and reduced in size with a mass of scirrhus extending from the pyloric to the cardiac end of the stomach, which when cut through was very resistent, grating under the scalpel, of a gray colour, with a dark deposit in the centre, and upon careful examination of the surface of the tumour there were several ulcerated patches.

The pancreas was firmly attached to the stomach so as to be with difficulty dissected from it.

In the cavity of the peritoneum were six pints of straw coloured fluid, having a disagreeable odour.

The whole of the intestines were glued together by bands of organized lymph, filled with soft and hard tubercles—these bands being with great difficulty torn so as to permit the other organs to be examined.

The transverse colon was bound fast to the peritoneum and the mesentery; glands were filled with the same deposit, a few of the tubercles being softened.

The liver and gall bladder were so firmly adherent to the walls of the abdomen, that they could not be readily removed; a portion of the liver removed, was of a dark red colour, with a hard deposit on its outer surface, with numerous bands of gray scirrhus matter obstructing the circulation, and thus causing the dropsical effusion. The spleen was softened and congested; no deposit in its substance, but it was tied down by adhesions like all the other organs.

The kidneys were very much enlarged, but healthy. The bladder was firmly adherent to the rectum, and much reduced in size, and the mucous membrane was softened and deeply congested.

*Remarks.*—There is a great difference of opinion as to the cause of cancer of the stomach. By M. Andral the disease is classified with chronic inflammation of the organ. Broussais and his followers believe that cancer is a consequence of inflammatory action. Breschet and Ferras also maintain that it succeeds to irritation, while Adams and Carmichael believed it to be a living being enjoying independent existence.

Cruveilhier, Lobstein, Carswell, and Budd, all think that it is a lesion of secretion or nutrition, and we have as many opinions as there are distinguished pathologists.

If there be a cancerous diathesis, and that according to Budd and Carswell the cancerous matter exists in the blood of such an individual, it appears that a very slight cause, as intemperance, as in the case related, will produce its development more in one organ than another; but ultimately it is distributed by the blood all over the body, and there is no organ that has been exempted from its pernicious influence.

*Diagnosis.*—The difficulties in the way are very great in making a correct diagnosis, especially when the tumour is situated on the posterior portion of the stomach. It has been often confounded with chronic gastritis and dyspepsia, and even the acid eructations, vomiting of brownish matter, with the lancinating pains, have all been seen by Andral in a case where the autopsy revealed nothing but chronic gastritis, and he declares that except in those cases where a tumour can be felt, there is no certain sign to distinguish the one affection from the other.

*Treatment.*—Various remedial means have been used in treatment of this disease, but few remedies exert much influence upon it; and the only two agents which we have to resort to, are arsenic and iodine; the one having been used for ages and the other being of later introduction; and when the one or the other is united with the bicarbonate of potassa, it increases its power, for according to M. Trousseau, patients have been cured by him, presenting all the rational symptoms of commencing scirrhus, and the waters of Vichy which contain it in solution, are very much used in France for the cure of this disease. But when no cure is even to be hoped for, the only thing for the humane physician to do is to make the patient's passage to the grave as easy as possible, by giving large doses of morphia, extract of hemlock, hyoscyamus, or other powerful sedatives which allay pain, and cause the patient to sleep away his life unconscious of the suffering which he endures.

*October*, 1847.

